# Effects of opium on hippocampal-dependent memory, antioxidant enzyme levels, oxidative stress markers, and histopathological changes of rat hippocampus

**DOI:** 10.1016/j.bbrep.2025.101993

**Published:** 2025-04-01

**Authors:** Ebrahim Abbasi, Fatemeh Mirzaei, Sodabeh Mashayekhi, Iraj Khodadadi, Alireza Komaki, Nafiseh Faraji, Seyed Alireza Vafaii

**Affiliations:** a Nutrition Health Research Center, Institute of Health Sciences and Technologies, Hamadan University of Medical Sciences, Hamadan, Iran; b Research Center for Molecular Medicine, Institute of Cancer, Hamadan University of Medical Sciences, Hamadan, Iran; c Department of Biochemistry, School of Medicine, Kermanshah University of Medical Sciences, Kermanshah, Iran; d Neurophysiology Research Center, Institute of Neuroscience and Mental Health, Hamadan University of Medical Sciences, Hamadan, Iran

**Keywords:** MDA, Opium, Learning, Memory

## Abstract

**Background:**

Opium addiction can affect various organs such as the liver, intestine, kidney, and brain. The hippocampus is one of the brain regions affected early on in Alzheimer's disease and has a vital role in neurogenesis, cognitive function, and memory. This region also is sensitive to oxidative stress and pathophysiological alterations. Hence, this study evaluated the effects of opium on memory and learning, and oxidative stress in the hippocampus of male addicted rats. Since, the hormonal alterations in female arts can affect immune response, metabolism, and behavior, we have selected male rats.

**Methods:**

Male rats were randomly divided into two groups: control and opium addicts. Animals received opium (40 mg/kg) for one month. Then, naloxone (2 mg⁄kg), a morphine antagonist, was injected intraperitoneally to confirm addiction. The activities and gene expressions of glutathione peroxidase (GPX), glutathione reductase (GPr), and superoxide dismutase (SOD) were determined by ELISA and Real-time PCR, respectively. Total antioxidant capacity (TAC), total oxidative state (TOS), glutathione, and malondialdehyde (MDA) concentrations, as well as hippocampus histopathology were assessed. Memory and learning were determined by water maze shuttle box tests.

**Results:**

The TAC and glutathione levels were decreased, while MDA and TOS increased (*P* < 0.05) in addicted animals. The gene expressions and activities of GPX, GPr, and SOD decreased in opium-treated animals when compared to control (*P* < 0.05). Histological analysis showed structural changes in the hippocampal in the opium group. Opium also impaired memory and learning in animals (*P* < 0.05).

**Conclusion:**

Opium consumption has a detrimental effect on hippocampus function and structure.

## Highlights

• Opium reduces antioxidant enzyme gene expression and activities in the brain
• Opium increases oxidative stress markers in the brain
• Opium impairs passive avoidance learning
• Opium impairs memory and spatial learning
• Opium leads to histopathological changes in the brain

## 1 Introduction

Papaver somniferum L. (opium poppy) is one of the well-known herbal medicines that has more than 80 different types of alkaloids [1]. The main alkaloids of opium, include morphine, thebaine, papaverine, codeine, etc. [2]. Since thousands of years ago, opium has been administered for suppressing the central nervous system (CNS) [3].

Today, opium addiction is one of the most common forms of drug abuse in the world. Opium addiction is common in the Middle East. Some people believe that opium improves blood sugar and lipids, and this is one of the motivations for people to use opium, especially in conditions of hypercholesterolemia, diabetes, etc. In recent years, the prevalence of opium abuse and dependence among young people and adolescents has increased. Due to the hidden nature of this phenomenon, accurate statistics on the prevalence of opium abuse and dependence are not available [4].

Addiction can lead to major public concerns, along with significant economic, social, and health consequences [5]. It has been shown that a substantial portion of the problems associated with addiction are the neuropathological changes in the CNS [6,7]. These neurological alterations range from anatomical to molecular-scale disturbances in different brain parts. As a result of opium-associated neural alterations, addicted subjects have deficits in various domains of cognitive functioning, including planning, inhibition, memory, and behavioral regulation [8]. Furthermore, opium has been shown to increase oxidative stress. Oxidative stress is one of the main contributing factors in neurodegenerative disorders. Previous studies reported that opium can lead to oxidative stress [9,10]. When brain cells are attacked by ROS, body antioxidant systems such as glutathione reductase (GR), glutathione peroxidase (GPX), and superoxide dismutase (SOD) will gradually reduce because of excessive consumption. On the other hand, the malondialdehyde (MDA) increased, which can reflect the oxidative damage. Increased MDA concentration and decreased total antioxidant, glutathione levels, and antioxidant enzyme activity (especially SOD, etc.) in addicted patients may play an important role in the progression of various diseases such as neurological disorders [11].

Due to high metabolic demands, low concentration of endogenous scavengers (e.g., GPx, SOD, vitamin C, catalase, etc.), extensive dendritic and axonal networks, high energy use, and high content of proteins and lipids is vulnerable to oxidative stress damage [12]. Inside the cell, free radicals such as hydroxyl (OH^−^), superoxide (O_2_^-^), hydrogen peroxide (H_2_O_2_), and peroxynitrite (ONOO^−^) are capable of producing free radicals. Free radical attack causes damage to cells by oxidizing proteins, membrane lipids, and DNA [11].

The body removed free radicals by cellular antioxidant enzymes, including GPr, GPx, and SOD [13]. Therefore, the aim of this study was to investigate the effect of opium on oxidative stress, inflammation, memory, and learning in Wistar rats.

## 2 Method and materials

### 2.1 Animals

In this study, 8-week-old male Wistar rats with an average weight of 200-220 were purchased from Hamadan University of Medical Sciences (Hamadan, Iran). The animals were kept in the animal room for 10 days to adapt to the new environment with the same standard food and in a natural dark-light cycle and proper ventilation. The animals were randomly divided into 2 groups: 1) the control group, and 2) the addicted group received 40 mg/kg of opium orally daily for one month. Addiction induction was performed using previous studies [14]. Naloxone (2 mg⁄kg), a morphine antagonist, was injected intraperitoneally (i.p.) into one rat/group to confirm addiction. After naloxone injection rats show behavioral alterations such as head and wet-dog shaking, writhing, crawling, chewing, jumping, ptosis, paw tremor, teeth chattering, diarrhea, and runny nose [15]. The animals that received naloxone were excluded from the study.

After one month, memory and learning tests were performed and then the rats were anesthetized with ketamine (75 mg)/xylazine (10 mg/kg), and blood was taken from the hearts of the animals. Blood samples were centrifuged at 3000 g for 5 min to prepare serum. The serum was stored at −20 °C until the day of the experiment. The hippocampus of the animals was separated immediately after killing the rats, frozen in liquid nitrogen, and then stored at −80 °C until gene expression studies were performed. The hippocampus was homogenized in phosphate buffer containing protease inhibitors and then centrifuged at 9000 rpm for 20 min and the supernatant was separated and used for antioxidant activity. Four rats from each group were also used to examine histological changes.

### 2.2 Water maze

This experiment used a water maze apparatus to examine spatial memory. This device consists of a black circular pool with a diameter of 1.8 m and a height of 60 cm, filled with water to a height of 25 cm. This tank is divided into four quadrants, and a small platform with a diameter of 1 cm is placed below the water surface in the center of one of the four quadrants, where the animal can rest. The rats were put in the water at one of the four randomly chosen quadrants and the time between entry into the water and escape onto the platform (escape latency) was determined. In this study, the platform was placed in one of the quadrants during the three-day experiment. The movement of each rat is monitored by a camera (Nikon, Melville, NY). Each rat was examined for 3 days based on the previously published paper. Three criteria were used to assess spatial memory, including 1) Escape latency, 2) Traveled distance, and 3) Spent time in the target quadrant [16].

### 2.3 Shuttle box

The shuttle box is a device for conducting behavioral experiments and passive avoidance learning tests. It consists of dark and lighted compartments, which are 20 × 20 × 30 cm. A guillotine door that the researcher can close or open separates the box. An electric shock generator was placed on the floor of the dark box. The training test was performed according to the previously published paper. The rats' long-term memory performance was evaluated on the second or test day, 24 h after the training phase. The animal was put in the lighted part, the guillotine door was closed after 5 s, and the time spent in the dark compartment (TDC) and the step-through latency in the retention test (STLr) were recorded. The test phase was finished when the rats either entered the dark compartment or stayed in the lighted box for 300s, demonstrating retention of the passive avoidance response. The electric shock was not applied during the retention test on the test day [16].

### 2.4 Total antioxidant capacity (TAC)

TAC levels in hippocampus lysate were determined using colorimetric kits according to the manufacturer's instructions (KTAC96, Kiazist, Iran). In this method, cupric (Cu^2+^) is reduced to cupro (Cu^+^) in the presence of antioxidants and produces color. The absorbance of the produced color was measured at 450 nm by an ELISA reader. Briefly, the hippocampus sample was homogenized in PBS buffer and centrifuged at 12,000 rpm for 15 min. The supernatant was combined with a TAC working solution and incubated for 45 min. Then the absorbance was measured at 450 nm. The total antioxidant capacity of the samples was calculated using a standard curve and reported as nmol of Trolox equivalent/mg protein [17].

### 2.5 Total oxidative state (TOS)

The total oxidative state (TOS) indicates the total free oxygen species and oxidative stress. According to the manufacturer's instructions, TOS in hippocampus lysates was measured using colorimetric kits (KTOS-96, Kiazist, Iran). In this reaction, ferrous Fe^2+^ is oxidized to ferric Fe^3+^ in the presence of oxidants, producing a color. The absorption rate is directly related to the amount of oxidant and the standard curve is drawn in the presence of H_2_O_2_. Briefly, a small portion of hippocampus tissue was homogenized in PBS buffer and centrifuged at 6000g for 15 min. The supernatant was added to the TOS solution and incubated for 15 min. The absorbance of the sample was read at 560 nm [17].

### 2.6 Measurement of lipid peroxidation (MDA)

Oxidative stress induces the peroxidation of unsaturated fatty acids, forming various aldehydes, especially MDA. Malondialdehyde in hippocampus lysates was measured using colorimetric kits according to the manufacturer's instructions (KMDA-96, Kiazist, Iran). Briefly, the sample was homogenized with the recommended amount of MDA Lysis Buffer and BHT. Then, the sample was centrifuged for 10 min at 6000g, and the supernatant was separated, and incubated with TBA solution for 60 min at 95 °C. The absorbance of the product was measured at 532 nm.

### 2.7 Superoxide dismutase (SOD) activity

The activity of SOD was measured in hippocampus lysate using colorimetric kits according to the manufacturer's instructions (ZellBio Cat. No: ZB-SOD-96A). SOD is effective in preventing oxidative stress by neutralizing superoxide ions. This method converted superoxide anion to hydrogen peroxide and oxygen under an enzymatic reaction. The colorful products were measured calorimetrically at a wavelength of 570 nm. The activity of SOD was expressed as the percentage of inhibition. The SOD activity was reported as mU/mg protein.

### 2.8 Glutathione reductase (GPr) and glutathione peroxidase (GPX) activities

The activities of glutathione peroxidase (GPx) and glutathione reductase (GPr) in hippocampus lysates were measured using colorimetric kits according to the manufacturer's instructions (Zellbio, Germany with Cat. No: ZB-GPX-A96). GPx detoxifies peroxides in the body and protects the hippocampus from oxidative damage. This enzyme also catalyzes the reduction of organic peroxides (R-*O*-O-H) and H_2_O_2_ to stable alcohols (R-*O*-H) and water. Glutathione is oxidized by GPx and then recycled to reduce glutathione by glutathione reductase (GPr) with the oxidation of NADPH to NADP^+^. The reduction in absorbance indicates the oxidation of NADPH to NADP^+^ which directly shows the GPx activity. The absorbance of the sample was measured at 340 nm and the results were expressed as mU/mg protein.

### 2.9 Real time-PCR

RNA extraction was performed using Trizol reagent (Invitrogen Co. Catalog # 15596-026). The concentration of RNA obtained was measured using a nanodrop device. The optical absorption ratio of this material was determined at a wavelength of 260 and 280 nm and the results showed that the RNA was of appropriate purity for the sample. Also, the quality of RNA was checked using agarose gel. cDNA was constructed using the available kits (GeneAll Biotechnology Co., Ltd.). Real-time PCR was performed using a real-time cycler (Roche) and an Ampliqon Real Q master mix (Ampliqon, Odense-Denmark, Cat. No.: A313402). This kit contains the necessary PCR components as a Master Mix. This device measured the expression level of target genes relative to the control gene as a housekeeping gene. Primers were used using previous articles. The available kit (Amplicon) uses SYBER Green, a fluorescent dye that emits fluorescence after binding to double-stranded DNA and can be measured by a Real-Time device [18].

### 2.10 Histological examination

The hippocampus (n=4) was immediately removed and fixed in 10 % formalin for histological studies. Paraffin sections and 5-μm-thick sections were prepared. The sections were stained with hematoxylin-eosin (H&E) and checked by light microscopy (Olympus, Tokyo, Japan). Morphological alterations in the hippocampus were reported based on cellular and structural changes.

### 2.11 Statistical analysis

In this study, the statistical analyses were done using GraphPad Prism software (version 8.0.2). Data are expressed as mean ± standard deviation (SD). Student's t-test was conducted to determine the difference between addiction and healthy groups. Two-way analysis of variance (ANOVA) was used to analysis the memory and learning tests. A P value less than 0.05 was considered as a statistically significant.

## 3 Results

### 3.1 Spatial memory

**Fig. 1:**
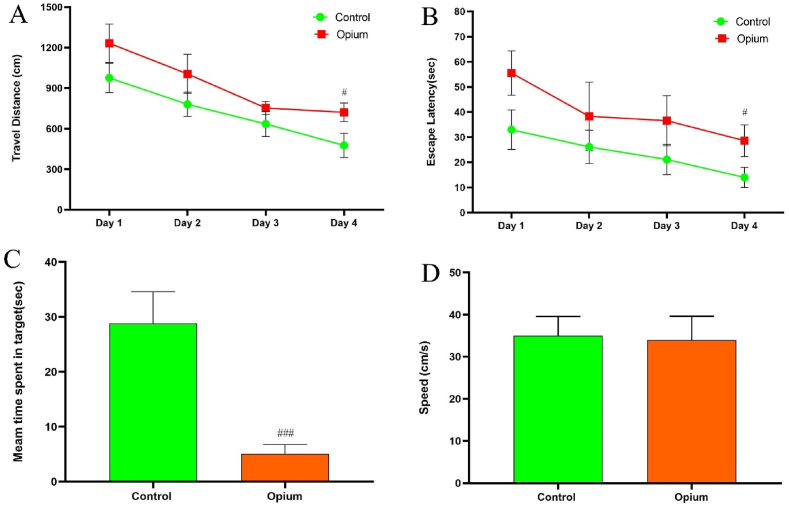
Effects of opium on spatial learning and memory. Addicted animals showed reduced traveled distance (A), escape latency time(B), and time spent in the target quarter (C) when compared to control rats. Data are expressed as mean ± SD. ^#^P < 0.05 and ^###^P < 0.001 compared to the control group.

Our results show a significant reduction of memory and spatial learning in addicted animals by evaluating escape latency and traveled distance between addicted and control groups. The escape latency in the control group was significantly less than the addicted group (*P* < 0.05). The travel distance also significantly increased in addicted animals when compared to the control (*P* < 0.05, Fig. 1).

The time spent in the target quadrant was notably reduced in the addicted group compared to the control rats, indicating that opium affected spatial memory (*P* < 0.001, Fig. 1). Our result showed that there was no significant difference in swimming speed between addiction and normal groups. This indicates that animals in both groups learned the task (Fig. 1).

### 3.2 Passive avoidance learning

**Fig. 2:**
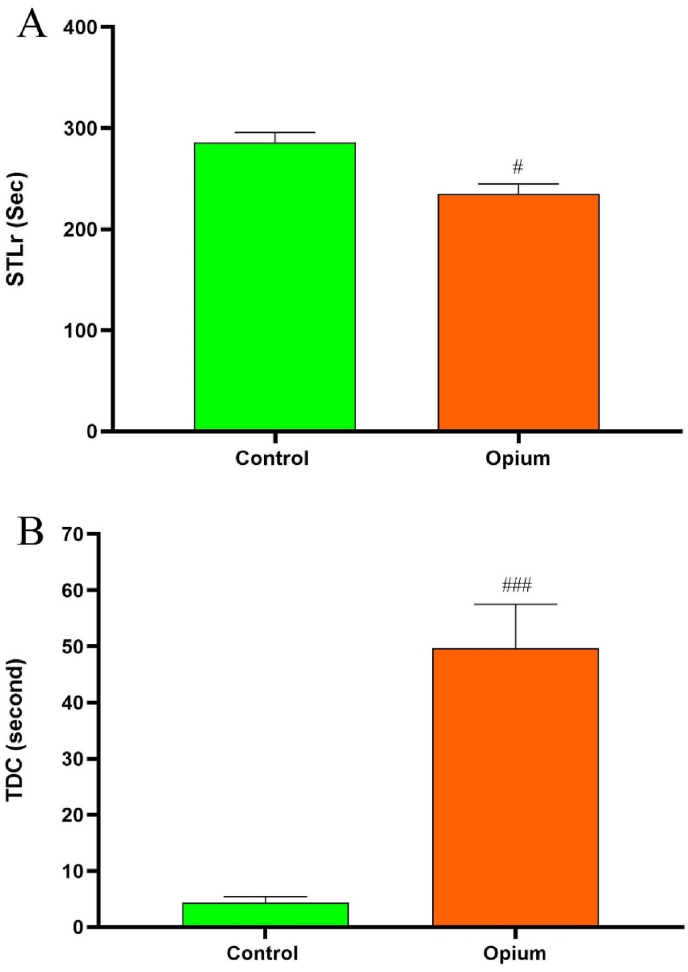
Effects of opium on passive avoidance learning. The results of the passive avoidance task show that addicted animals showed reduced step through latency (STL) and increased time in the dark compartment (B) compared to control rats. Data are expressed as mean ± SD. ^#^P < 0.05 and ^###^P < 0.001 compared to the control group.

In the passive avoidance learning test (shuttle box) the time spent in the dark compartment (TDC) and the step-through latency in the retention test (STLr) were determined. An ANOVA analysis showed that the STLr was significantly reduced in addicted animals compared to the control rats (*P* < 0.05). Our results also show a significant difference in the TDC between both groups. TDC in the addicted animals was significantly more than control group rats (*P* < 0.001, Fig. 2).

### 3.3 Oxidative stress markers

**Fig. 3:**
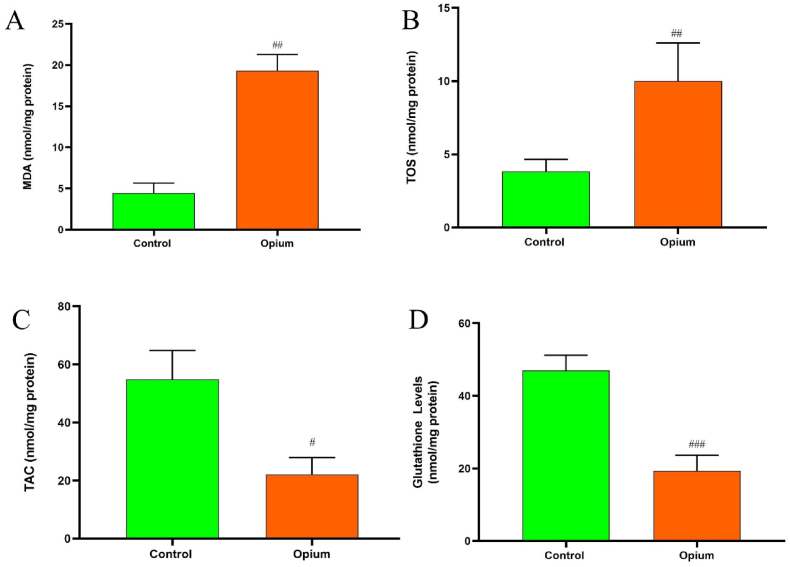
Effects of opium on oxidative stress markers. The MDA and TOS were increased and TAC and glutathione were reduced in addicted animals compared to control rats. Data are expressed as mean ± SD. ^#^P < 0.05, ^##^P < 0.01 and ^###^P < 0.001 compared to the control group. MDA: Malondialdehyde, TAC: Total antioxidant capacity, TOS: Total oxidative state.

The statistical analysis indicated a significant difference in the levels of MDA in different treatment groups (*P* < 0.01). MDA, a byproduct of lipid peroxidation, significantly increased in the hippocampus of addicted rats compared with the normal group (*P* < 0.01, Fig. 3).

TOS is a key factor for determining oxidative stress, which was increased significantly in addicted rats compared to the control group (*P* < 0.01, Fig. 3).

Opium administration also significantly reduced TAC levels in the addicted rats compared to control rats (*P* < 0.05). In line with these results, GSH concentration was also significantly reduced in the hippocampus of addicted rats compared to the control rats (*P* < 0.001, Fig. 3).

### 3.4 Antioxidant enzyme gene expressions

**Fig. 4:**
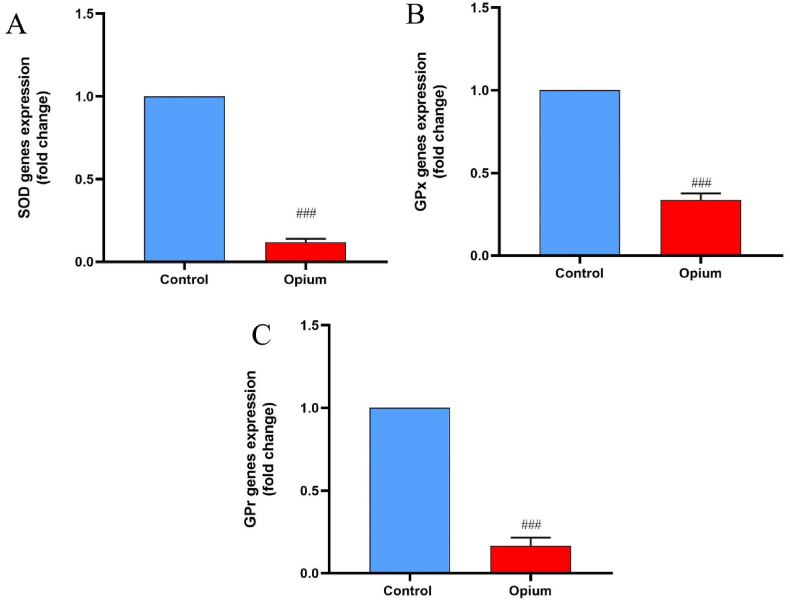
Effects of opium on the gene expression of antioxidant enzymes. The expressions of SOD (A), GPX (B), and GPr (C) were reduced in addicted animals compared to control rats. Data are expressed as mean ± SD. ^###^P < 0.001 compared to the control group. SOD: superoxide dismutase, GPX: glutathione peroxidase, GPr: glutathione reductase.

Gene expression of SOD in the hippocampus of addicted animals was reduced compared to the control group (*P* < 0.001, Fig. 3). Significant down-regulation of GPx and GPr was detected in addicted animals compared to the control rats (P < 0.001, Fig. 4).

### 3.5 Antioxidant enzyme activities

**Fig. 5:**
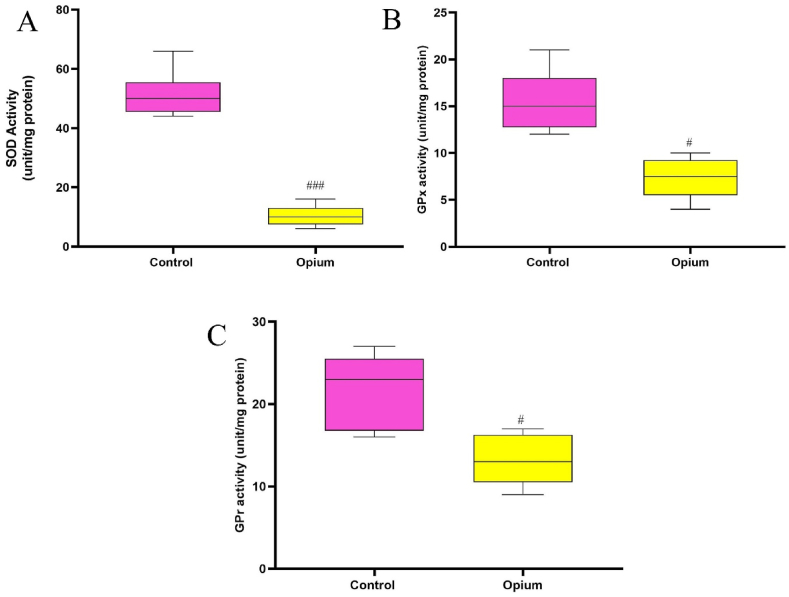
Effects of opium on the activity of antioxidant enzymes. The activities of SOD (A), GPX (B), and GPr (C) were reduced in addicted animals compared to control rats. Data are expressed as mean ± SD. ^#^P < 0.05 and ^###^P < 0.001 compared to the control group. SOD: superoxide dismutase, GPX: glutathione peroxidase, GPr: glutathione reductase.

Compared with the control rats, administration of opium significantly increased the SOD in the hippocampus (*P* < 0.001, Fig. 5).

Addicted animals showed a significant (*P* < 0.05) reduction in the activity of GPx and GPr in the hippocampus when compared with the control group (Fig. 5).

### 3.6 Histopathological findings

**Fig. 6:**
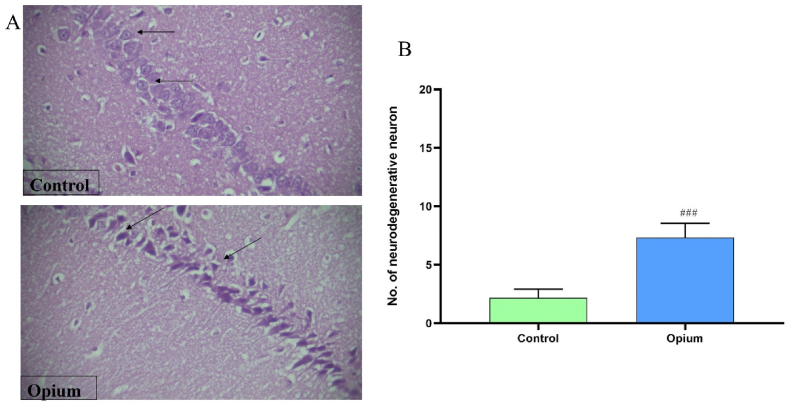
Effects of opium on CA1 region of the hippocampus. Opium changed the morphology CA1 of the hippocampus. The number of neurodegenerative neurons was also reduced in addicted animals (B). Data are expressed as mean ± SD. ^###^P < 0.001 compared to the control group.

Histological results show that the structure of the CA area of the hippocampus, the dentate gyrus, and the subiculum regions was normal. In the addicted group, pyramidal cells decreased in thickness, organized irregularly, and shrank. Other morphological alterations were observed, including necrosis, abnormal nuclei, disintegrated axons, and vacuolation. The number of neurodegenerative neurons was also reduced in addicted animals (*P* < 0.001, Fig. 6).

## 4 Discussion

In this study, rats that received opium orally had a significant increase in MDA and TOS levels and a significant decrease in glutathione and TAC levels. Previous studies reported that opium may cause tissue damage through mitogenesis, oxidative DNA damage, and chromosomal damage [19,20].

Opium or its metabolites, such as morphine, cause tissue damage by increasing of MDA, TOS ad reducing TAC and glutathione levels [21]. Free radicals cause protein denaturation, DNA degradation, and lipid peroxidation. These events lead to cell membrane damage and increase the reactive aldehydes such as MDA. MDA is a useful indicator for demonstrating lipid peroxidation, which can ultimately lead to memory and learning impairment. The significant increase in MDA concentration and the decrease in antioxidant enzyme activity in addicted individuals may play an important role in the development of its dangerous complications because the increase in MDA and decrease in brain antioxidant levels lead to neuronal destruction [10]. In line with our study, Bakhshayesh et al., [22] Ghazavi et al., [23] reported that opium reduces antioxidant enzyme activities and increases the production of free radicals. It has been proven that free radicals such as superoxide, hydroxyl radical, oxygen peroxide, and radicals generated by lipid peroxidation cause damage to nervous tissue and cause neurological diseases.

It has been reported that morphine causes a decrease in glutathione and causes hepatotoxicity and neurotoxicity. Studies have shown that morphine depleted glutathione levels and an increase in the production of free radicals [24]. Long-term use of opium or its alkaloids can be associated with some pathological consequences, including neurological disorders, hepatotoxicity, renal dysfunction, oxidative stress, and apoptosis [20,23]. Bhat et al. showed that morphine induces oxidative stress and also increases apoptosis in macrophages [25].

It has been established that there is a direct relationship between neurodegenerative diseases and oxidative stress which is considered by the reduced activity of antioxidant enzymes such as CAT, GPx, and SOD [10]. In Alzheimer's disease (AD) animal models, up-regulation of SOD reduces memory deficits by decreasing superoxide in the hippocampus [26].

Our study showed that opium reduced the activity of GPx, GPr, and SOD in the hippocampus. SOD has a vital role in catalyzing the of reactive superoxide (O_2_^-^) to hydrogen peroxide (H_2_O_2_) which is less reactive. GPx is another antioxidant enzyme that catalyzes the reduction and lipid peroxides and hydrogen peroxide utilizing glutathione. GPx is located in both mitochondria and cytosol. CAT also converts hydrogen peroxide to oxygen and water using either manganese or iron as a cofactor. This enzyme is located in mitochondria, cytoplasm, and peroxisomes. The function of CAT is minor at low levels of hydrogen peroxide, while it becomes more important at higher concentrations of hydrogen peroxide. Glutathione participates in two reactions, including reactions with ROS such as superoxide radical and hydroxyl radical, and acts as an electron donor for the reduction of GPx [27]. Increased oxidative stress may increase the production of inflammatory cytokines through several mechanisms. Reactive oxygen species act as second messengers and activate the transcription of inflammatory cytokine genes [28]. Previous studies have also shown that opium causes inflammation in various tissues [23,29,30]. Peng et al. showed that morphine administration to mice increased TNF-α and interleukin 12 [31]. Asgari et al., by examining some inflammatory factors in addicted and healthy individuals, showed that the levels of HbA1c, fibrinogen, factor VII, and C-reactive protein (CRP) were higher in opium users than in healthy individuals [32]. Asadi Karam et al., by administering opium. Asadikaram et al., showed that opium increased the pro-inflammatory cytokines INF-gamma TNF-α while reducing the secretion of anti-inflammatory cytokines such as interleukin 10 (IL-10) and IL-4 [33].

Naderi et al., [34] also showed that opium administration increases systolic and diastolic blood pressure, fibrinogen, and inflammation in the blood. Zhang et al. showed that morphine administration to mice reduces catalase and glutathione peroxidase activity, reduces glutathione and antioxidant levels, and increases malondialdehyde in the liver [35].

It has been reported that both chronic and acute administration of morphine affect memory and learning. Morphine injection impaired exploratory behavior and spatial memory function in both the Y-maze and Morris Water Maze tests [36]. In this study, memory and learning in the addicted group were significantly reduced. Oxidative stress plays a significant role in the memory and learning process. Previous studies show that opium reduces memory and learning [36]. Increased oxidative stress in the brain plays an important role in cognitive impairment and neurological diseases [9].

Oxidative stress is known as a main factor in the development and progression of neurodegenerative diseases such as Parkinson's disease (PD) and AD. Increased free radicals damage cellular components such as lipids, proteins, and DNA, in the brain, causing neural death. Oxidative stress is involved in cognitive decline and memory loss as observed in AD subjects [27]. MDA causes brain damage and also memory and learning impairment. Lipid oxidation products such as MDA can react with biomolecules and show cytotoxic and neurotoxic effects. Therefore, the high presence of free radicals, especially compounds resulting from lipid peroxidation, plays an important role in reducing memory and learning [9,10]. The exact mechanisms involved in morphine-induced memory loss are not yet fully understood. Nevertheless, changes in synaptic plasticity and neurotransmitter function can lead to memory impairment [6]. Sarkaki et al., reported that morphine addiction in parents led to memory deficiency via the decrease of long-term potentiation (LTP) in the hippocampus [37]. Prenatal opioid exposure affects spine formation and dendritic growth in the brains of rats (17). Morphine also impairs the spatial memory and learning (18). Sepehri et al., also reported that opium addiction of both parents reduced the memory and learning process in their offspring [38].

In this study, opium also caused histopathological changes in the hippocampus, which are in line with antioxidant changes. Bakhshayesh et al., administrated 30, 75, and 100 μl *Opium Tincture* to rats for 21 days. They results show that Opium Tincture reduced the granular layer thickness of the dentate gyrus, and the number of immature and mature neurons [7]. Iranpour et al., by examining the effect of morphine on the histopathological structure of the adult rat brain, showed that morphine increased the number of astrocytes, increased the count of glial scars, and induced necrosis in the brain [39].

## 5 Conclusion

In conclusion, the findings of this experiment showed that the gene expressions and activities of GPx, GPr, and SOD were reduced by opium in the hippocampus. Furthermore, opium reduced TAC, and glutathione levels and increased MDA and TOS. Histological analysis showed that opium had deteriorating effects on the hippocampus. Opium also impairs memory and learning in addicted animals. This study has demonstrated that opium consumption has a detrimental impact on hippocampus function and histology.

## CRediT authorship contribution statement

**Ebrahim Abbasi:** Writing – review & editing, Writing – original draft, Visualization, Software, Resources, Methodology, Investigation, Funding acquisition, Formal analysis, Data curation, Conceptualization. **Fatemeh Mirzaei:** Writing – review & editing, Visualization, Validation, Project administration, Methodology, Investigation. **Sodabeh Mashayekhi:** Visualization, Validation, Software, Resources, Methodology, Investigation, Conceptualization. **Iraj Khodadadi:** Writing – review & editing, Visualization, Validation, Supervision, Funding acquisition, Formal analysis. **Alireza Komaki:** Visualization, Validation, Supervision. **Nafiseh Faraji:** Methodology, Investigation. **Seyed Alireza Vafaii:** Software, Resources, Methodology, Investigation, Data curation.

## Ethics approval and consent to participate

This study was completed in accordance with the guidelines of the arrive declarations and carried out in accordance with guidance on the operation of the animals (Scientific Procedures) Act 1986 and associated guidelines. All processes of this study were approved by the ethical committee of Hamadan Medical University Sciences (Hamadan, Iran), based on the guidelines of laboratory animals (ethic code: IR.UMSHA.REC.1396.794).

## Funding

This study was funded by the 10.13039/501100004697Hamadan University of Medical Sciences (No: 9612087763).

## Declaration of competing interest

The authors declare the following financial interests/personal relationships which may be considered as potential competing interests: Ebrahim Abbasi reports financial support was provided by 10.13039/501100004697Hamadan University of Medical Sciences. If there are other authors, they declare that they have no known competing financial interests or personal relationships that could have appeared to influence the work reported in this paper.

## Data Availability

Data will be made available on request.

## References

[1] Kumar B., Patra N.K. (2012). Inheritance pattern and genetics of yield and component traits in opium poppy (Papaver somniferum L.). Ind. Crop. Prod..

[2] Carlin M.G., Dean J.R., Ames J.M. (2020). Opium alkaloids in harvested and thermally processed poppy seeds. Front. Chem..

[3] Salavert A., Zazzo A., Martin L., Antolín F., Gauthier C., Thil F., Tombret O., Bouby L., Manen C., Mineo M., Mueller-Bieniek A., Piqué R., Rottoli M., Rovira N., Toulemonde F., Vostrovská I. (2020). Direct dating reveals the early history of opium poppy in western Europe. Sci. Rep..

[4] Roayaei P., Aminorroaya A., Vasheghani-Farahani A., Oraii A., Sadeghian S., Poorhosseini H., Masoudkabir F. (2020). Opium and cardiovascular health: a devil or an angel?. Indian Heart J..

[5] Lo T.W., Yeung J.W.K., Tam C.H.L. (2020). Substance abuse and public health: a multilevel perspective and multiple responses. Int. J. Environ. Res. Publ. Health.

[6] Bayassi-Jakowicka M., Lietzau G., Czuba E., Patrone C., Kowiański P. (2022). More than addiction-the nucleus accumbens contribution to development of mental disorders and neurodegenerative diseases. Int. J. Mol. Sci..

[7] Bakhshayesh A., Eslami Farsani R., Seyedebrahimi R., Ababzadeh S., Heidari F., Eslami Farsani M. (2023). Evaluation of the negative effects of opium tincture on memory and hippocampal neurons in the presence of chicory extract. Adv. Biomed. Res..

[8] Sanjari Moghaddam H., Shadloo B., Shahkhah H., Tafakhori A., Haghshomar M., Meshkat S., Aghamollaii V. (2021). Cognitive impairment in opium use disorder. Behav. Neurol..

[9] Dash U.C., Bhol N.K., Swain S.K., Samal R.R., Nayak P.K., Raina V., Panda S.K., Kerry R.G., Duttaroy A.K., Jena A.B. (2024). Oxidative stress and inflammation in the pathogenesis of neurological disorders: mechanisms and implications. Acta Pharm. Sin. B.

[10] Lee K.H., Cha M., Lee B.H. (2020). Neuroprotective effect of antioxidants in the brain. Int. J. Mol. Sci..

[11] Gusti A.M.T., Qusti S.Y., Alshammari E.M., Toraih E.A., Fawzy M.S. (2021). Antioxidants-related superoxide dismutase (SOD), catalase (CAT), glutathione peroxidase (GPX), glutathione-S-transferase (GST), and nitric oxide synthase (NOS) gene variants analysis in an obese population: a preliminary case-control study. Antioxidants.

[12] Dorman D.C. (2023). The role of oxidative stress in manganese neurotoxicity: a literature review focused on contributions made by professor michael aschner. Biomolecules.

[13] Slimani W., Maioli M., Cruciani S., Zerizer S., Santaniello S., Kabouche Z., Coradduzza D., Chessa M., Fancello S., Migheli R., Serra P.A., D'Hallewin G. (2023). Antioxidant, anti-inflammatory and anti-proliferative properties of stachys circinata on HepG2 and MCF7 cells. Plants.

[14] Mohammadi A., Oshaghi E.A., Sorkhani A.N., Oubari F., Kia R.H., Rezaei A. (2012). Effect of opium on lipid profile and expression of liver X receptor alpha (LXRα) in normolipidemic mouse. Food Nutr. Sci..

[15] Zeinalinejad H., Ramezani M.A., Shafiee M., Karvar M., Malekpour-Afshar R. (2011). Effect of opium dependency on burn healing in a rat model: an experimental study. Med. Princ. Pract..

[16] Mohammadali S., Heshami N., Komaki A., Tayebinia H., Abbasi Oshaghi E., Karimi J., Hashemnia M., Khodadadi I. (2020). Dill tablet and Ocimum basilicum aqueous extract: promising therapeutic agents for improving cognitive deficit in hypercholesterolemic rats. J. Food Biochem..

[17] Mazloomi S., Sanoeei Farimani M., Tayebinia H., Karimi J., Amiri I., Abbasi E., Khodadadi I. (2022). The association of mitochondrial translocator protein and voltage-dependent anion channel-1 in granulosa cells with estradiol levels and presence of immature follicles in polycystic ovary syndrome. J. Reproduction Infertil..

[18] Abbasi-Oshaghi E., Khodadadi I., Tavilani H., Mirzaei F., Goodarzi M.T. (2018). Dill-normalized liver lipid accumulation, oxidative stress, and low-density lipoprotein receptor levels in high cholesterol fed hamsters. ARYA Atheroscler.

[19] Shabestari A.N., Tamehri Zadeh S.S., Fakhr Yasseri A., Ebrahimi M., Issa B., Rahimnia R. (2023). The impact of opium on men's fertility and DNA fragmentation: a literature review. Transl Res Urol.

[20] Abbasi E., Pourjafar M., Mirzaei F., Ghaleiha A., Ahmadi M., Mirzajani S. (2024). Effect of opium on oxidative stress markers in HepG2 cell line. Cell & Tissue J.

[21] Salehi I., Zarrinkalam E., Mirzaei F., Abbasi Oshaghi E., Ranjbar K., Asl S.S. (2018). Effects of resistance, endurance, and concurrent exercise on oxidative stress markers and the histological changes of intestine after morphine withdrawal in rats. Avicenna J Med Biochem.

[22] Bakhshayesh A., Farsani R.E., Seyedebrahimi R., Ababzadeh S., Heidari F., Farsani M.E. (2023). Evaluation of the negative effects of opium tincture on memory and hippocampal neurons in the presence of chicory extract. Adv. Biomed. Res..

[23] Ghazavi A., Mosayebi G., Solhi H., Rafiei M., Moazzeni S.M. (2013). Serum markers of inflammation and oxidative stress in chronic opium (Taryak) smokers. Immunol. Lett..

[24] Sadat-Shirazi M.-S., Zarrindast M.-R., Ashabi G. (2020). Oxidative stress enzymes are changed in opioid abusers and multidrug abusers. J. Clin. Neurosci..

[25] Bhat R.S., Bhaskaran M., Mongia A., Hitosugi N., Singhal P.C. (2004). Morphine-induced macrophage apoptosis: oxidative stress and strategies for modulation. J. Leukoc. Biol..

[26] Chidambaram S.B., Anand N., Varma S.R., Ramamurthy S., Vichitra C., Sharma A., Mahalakshmi A.M., Essa M.M. (2024). Superoxide dismutase and neurological disorders. IBRO Neurosci Rep.

[27] Kim G.H., Kim J.E., Rhie S.J., Yoon S. (2015). The role of oxidative stress in neurodegenerative diseases. Exp Neurobiol.

[28] Hong Y., Boiti A., Vallone D., Foulkes N.S. (2024). Reactive oxygen species signaling and oxidative stress: transcriptional regulation and evolution. Antioxidants.

[29] Momeni-Moghaddam M.A., Asadikaram G., Masoumi M., Sadeghi E., Akbari H., Abolhassani M., Farsinejad A., Khaleghi M., Nematollahi M.H., Dabiri S., Arababadi M.K. (2022). Opium may affect coronary artery disease by inducing inflammation but not through the expression of CD9, CD36, and CD68. J. Invest. Med..

[30] Purohit P., Roy D., Dwivedi S., Nebhinani N., Sharma P. (2022). Association of miR-155, miR-187 and inflammatory cytokines IL-6, IL-10 and TNF-α in chronic opium abusers. Inflammation.

[31] Peng X., Mosser D.M., Adler M.W., Rogers T.J., Meissler J.J., Eisenstein T.K. (2000). Morphine enhances interleukin-12 and the production of other pro-inflammatory cytokines in mouse peritoneal macrophages. J. Leukoc. Biol..

[32] Asgary S., Sarrafzadegan N., Naderi G.A., Rozbehani R. (2008). Effect of opium addiction on new and traditional cardiovascular risk factors: do duration of addiction and route of administration matter?. Lipids Health Dis..

[33] Asadikaram G., Igder S., Jamali Z., Shahrokhi N., Najafipour H., Shokoohi M., Jafarzadeh A., Kazemi-Arababadi M. (2015). Effects of different concentrations of opium on the secretion of interleukin-6, interferon-γ and transforming growth factor beta cytokines from jurkat cells. Addict Health.

[34] Naderi G., Asgary S., Sadeghi M., Sabetnezhad Z., Tansaz M. (2005). Comparing plasma level of CRP, factor VII, fibrinogen; platelet counts, systolic and diastolic blood pressure in smokers with opium addicted smokers. J Qazvin Univ Med Sci.

[35] Zhang Y.T., Zheng Q.S., Pan J., Zheng R.L. (2004). Oxidative damage of biomolecules in mouse liver induced by morphine and protected by antioxidants. Basic Clin. Pharmacol. Toxicol..

[36] Mohammadkhani M., Gholami D., Riazi G. (2024). The effects of chronic morphine administration on spatial memory and microtubule dynamicity in male mice's brain. IBRO Neuroscience Reports.

[37] Sarkaki A., Mard S.A., Bakhtiari N., Yazdanfar N. (2023). Sex-specific effects of developmental morphine exposure and rearing environments on hippocampal spatial memory. Int. J. Dev. Neurosci..

[38] Sepehri G., Parsania S., Hajzadeh M.A., Haghpanah T., Sheibani V., Divsalar K., Shekarforoush S., Afarinesh M.R. (2014). The effects of co-administration of opium and morphine with nicotine during pregnancy on spatial learning and memory of adult male offspring rats. Iran J Basic Med Sci.

[39] Iranpour M., Torkzadeh-Tabrizi S., Khatoon-Asadi Z., Malekpour-Afshar R. (2018). Immunohistochemical assessment of inflammation and regeneration in morphine-dependent rat brain. Addiction & Health.

